# Mechanical Properties of Silicon Nitride in Different Morphologies: In Situ Experimental Analysis of Bulk and Whisker Structures

**DOI:** 10.3390/ma17184549

**Published:** 2024-09-16

**Authors:** Bokang Wang, Tanglong Bai, Weide Wang, Hongti Zhang

**Affiliations:** 1 Shanghai Key Laboratory of High-Resolution Electron Microscopy, School of Physical Science and Technology, ShanghaiTech University, Shanghai 201210, China; 2 Science and Technology on Advanced Ceramic Fibers and Composites Laboratory, College of Aerospace Science and Engineering, National University of Defense Technology, Changsha 410073, China

**Keywords:** silicon nitride, mechanical property, size effect, nanoindentation, strain gradient plasticity

## Abstract

Silicon nitride (Si_3_N_4_) is widely used in structural ceramics and advanced manufacturing due to its excellent mechanical properties and high-temperature stability. These applications always involve deformation under mechanical loads, necessitating a thorough understanding of their mechanical behavior and performance under load. However, the mechanical properties of Si_3_N_4_, particularly at the micro- and nanoscale, are not well understood. This study systematically investigated the mechanical properties of bulk Si_3_N_4_ and Si_3_N_4_ whiskers using in situ SEM indentation and uniaxial tensile strategies. First, nanoindentation tests on bulk Si_3_N_4_ at different contact depths ranging from 125 to 450 nm showed significant indentation size effect on modulus and hardness, presumably attributed to the strain gradient plasticity theory. Subsequently, in situ uniaxial tensile tests were performed on Si_3_N_4_ whiskers synthesized with two different sintering aids, MgSiN_2_ and Y_2_O_3_. The results indicated that whiskers sintered with Y_2_O_3_ exhibited higher modulus and strength compared to those sintered with MgSiN_2_. This work provides a deeper understanding of the mechanical behavior of Si_3_N_4_ at the micro- and nanoscale and offers guidance for the design of high-performance Si_3_N_4_ ceramic whiskers.

## 1. Introduction

As a kind of advanced ceramic, silicon nitride (Si_3_N_4_) is widely used in the aerospace, mechanical engineering, and electronics industries due to its excellent mechanical, chemical, and physical properties, including high-temperature resistance, exceptional hardness, superior corrosion resistance, relatively high thermal conductivity, and electrical insulation [1,2,3]. To enhance its performance and service reliability in various applications, an in-depth understanding of its mechanical properties is crucial given that most device malfunctions are directly caused by the mechanical failure of their construction units. As structural reinforcement phases in composites, its primary covalently bonding nature leads Si_3_N_4_ to exhibit brittle fracture fashion with scarce deformability [4,5], which severely hinders its application in fields where a certain amount of toughness is required. Therefore, related research is imperative to examine the mechanical properties of Si_3_N_4_ at varied size scales and explore the corresponding deformability of Si_3_N_4_ ceramics.

Past works regarding the mechanical properties of bulk Si_3_N_4_ materials typically used indentation tests, dog-bone specimen tensile tests, micro-pillar compression, etc. Chakraborty et al. first characterized the microhardness values of bulk Si_3_N_4_ and discovered anisotropy in hardness, with the prismatic plane exhibiting higher hardness compared to the basal plane [6]. Subsequently, Dusza et al. found similar hardness anisotropy using a microhardness tester on gas-pressure-sintered β-Si_3_N_4_ polycrystalline grains, confirming that the prismatic plane has higher hardness than the basal plane [7,8]. However, their study was limited to only the prismatic and basal planes. Hay et al. used nanoindentation to determine the relationship between general orientation and hardness [9]. Later, Milhet et al. observed the dislocation structures after microhardness testing using transmission electron microscopy, identifying $\left\{10\bar{1}0\right\}$[0001] and $\left\{10\bar{1}1\right\}$<$1\bar{2}10$> slip systems, corresponding to prismatic and pyramidal slip, respectively [10]. In addition to hardness testing, Center et al. conducted creep studies on bulk Si_3_N_4_ samples under tensile and compressive loads [11]. They found that under tensile load, creep is linearly related to the applied stress at low stress levels and exponentially related at high stress levels. Under compressive conditions, creep is linearly related to stress with a slope of 1. With the advancement of micro- and nanotechnologies, Csanádi et al. first studied the mechanical properties of Si_3_N_4_ using micro-pillar compression experiments, finding that the yield stress is related to crystalline orientations and observing the activation of the $\left\{10\bar{1}0\right\}$[0001] slip system [12]. The most recent study on the mechanical properties of Si_3_N_4_ was conducted by Zhang et al., who performed nanoindentation tests on transparent silicon nitride and obtained hardness values close to those of traditional single crystals [13]. Recent studies have focused on enhancing the mechanical properties of silicon nitride by incorporating other materials to form silicon-nitride-based composites, aiming to improve both fracture toughness and hardness simultaneously [14,15]. For example, Chen et al. enhanced the hardness and fracture toughness of silicon nitride ceramics by sintering multilayer graphene and Si_3_N_4_ whiskers. Hardness was measured using a Vickers hardness tester, while fracture toughness was calculated using the Shetty formula [15]. Up to now, fundamental experimental research on the mechanical properties of bulk Si_3_N_4_, particularly its elastic modulus, is limited. Furthermore, for Si_3_N_4_ ceramics, which are extremely hard materials, there is a lack of experimental studies on the relationship between hardness and depth (i.e., the “indentation size effect”) and the relationship between hardness and elastic modulus.

With the continuous development of nanotechnology, Si_3_N_4_ at the micro- and nanoscale not only retains the high strength, high hardness, and excellent thermal stability of traditional materials but also exhibits significant optical, electrical, and biocompatibility characteristics, making it widely used in electronic and optical devices [16,17,18,19]. In the field of biomaterials, there has been little attention to the potential medical applications of silicon-based nanomaterials until now. In fact, Si_3_N_4_ in its bulk form has been shown to be biocompatible and stable in vivo, characteristics that, combined with its remarkable mechanical properties, make it an attractive ceramic implant material, particularly useful in some healthcare applications, especially in orthopedic surgery [20]. Shekaari et al. assessed the biocompatibility of 2D β-Si_3_N_4_ nanosheets by studying their potential interactions with human serum albumin (HSA) and the p53 tumor suppressor [21]. With the rise of organic bioelectronics, silicon nitride nanomaterials have found numerous applications in biosensors [22,23,24,25]. In the field of optoelectronics, silicon nitride as a third-generation semiconductor also has many applications in optoelectronic devices. In recent studies, Chen et al. designed a compact optoelectronic reversible logic gate based on a silicon nitride and potassium niobate hybrid nanofilm waveguide, which could improve digital circuit efficiency and potentially alleviate electronic bottlenecks [26]. Given its wide range of applications, understanding the mechanical properties of silicon nitride at the nanoscale is also of great importance. In terms of mechanical properties, adding Si_3_N_4_ whiskers to other ceramics or composite materials helps absorb residual energy during crack propagation when the material is damaged. The whiskers undergo debonding, pull-out, and fracture, altering the crack propagation path and consuming fracture energy, thus achieving reinforcement and toughening [27]. For example, Yang et al. found that Si_3_N_4_ whiskers can significantly improve the wear resistance and mechanical properties of Si_3_N_4_-based composites [28]. Subsequently, Lu et al. discovered that surface-modified β-Si_3_N_4_ whiskers can enhance dental resin composites [29]. Later studies also showed that Si_3_N_4_ whiskers can reinforce and toughen Si_3_N_4_/SiC ceramics and various other composites [30,31,32,33]. However, up to now, most studies have focused on characterizing the mechanical properties of ceramics mixed with Si_3_N_4_ whiskers. Experimental studies on the mechanical properties of the whiskers themselves have been scarce.

In this work, we studied the mechanical properties of two different kinds of silicon nitride at different sample geometries: bulk and whiskers. Using a nanoindenter, we performed in situ indentation experiments on bulk Si_3_N_4_ to measure its modulus and hardness to explore the “indentation size effect” and the relationship between modulus and hardness. Furthermore, in situ tensile tests were conducted on individual Si_3_N_4_ whiskers synthesized with two different sintering aids to analyze their Young’s modulus and fracture strength. Unlike existing studies, this paper systematically investigates the mechanical properties of bulk Si_3_N_4_ and Si_3_N_4_ whiskers synthesized with different sintering aids, providing new data to support a deeper understanding of the mechanical behavior of Si_3_N_4_.

## 2. Sample and Experimental Methods

In this work, the mechanical properties of bulk and whiskers, two forms of Si_3_N_4_ samples, were investigated. For bulk samples, the classical nanoindentation method was adopted to probe its mechanical properties, while for whisker samples, a device-assisted in situ nanomechanical stretching strategy was employed to evaluate their tensile properties. Both the nanoindentation and nanomechanical stretching tests were carried out inside a scanning electron microscope (SEM) under the ‘seeing is believing’ in situ approach [34,35].

### 2.1. Fabrication of Si_3_N_4_ Bulk Sample

The Si_3_N_4_ bulk sample was prepared using commercial α-Si_3_N_4_ (SN-E10, α phase > 95 wt.%, D50 = 0.5 μm, UBE Industries., Ltd., Tokyo, Japan) as the starting material. MgSiN_2_ (ZK-MGN, purity ≥ 99 wt.%, Technical Institute of Physics and Chemistry, Chinese Academy of Sciences, Beijing, China) and Y_2_O_3_ (D50 = 5 μm, purity ≥ 99.99 wt.%, Yuelong Chemical Co., Ltd., Yancheng, China) were used as sintering aids. In this work, the amount of sintering aids was fixed at 1.5 wt%. In order to achieve a homogeneous mixing, all powder mixtures were ball-milled in ethyl alcohol at a rotation speed of 300 rpm for 4 h. After drying at 80 °C for 12 h and sieving through a 100-mesh screen, the powder mixture was sintered at 1750 °C for 1 h, under a nitrogen pressure of 0.1 MPa.

### 2.2. Fabrication and Microstructural Characterization of Si_3_N_4_ Whiskers

**Figure 1:**
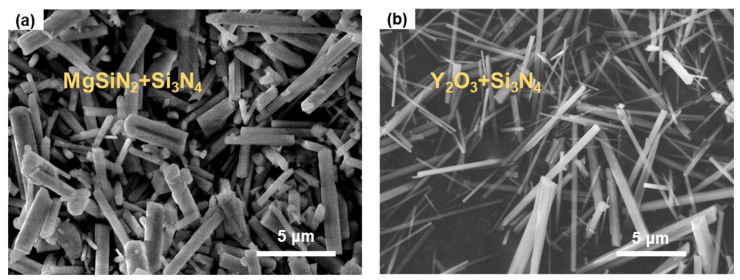
(**a**) SEM image of the MgSiN_2_+Si_3_N_4_ whiskers; (**b**) SEM image of the Y_2_O_3_+Si_3_N_4_ whiskers.

There are two types of silicon nitride whiskers. One type was synthesized by using MgSiN_2_ as the sintering aid and noted as MgSiN_2_+Si_3_N_4_ in this work. As shown in Figure 1a, the low-magnification SEM image of the sample, the MgSiN_2_+Si_3_N_4_ whiskers present smooth surfaces and uniform radial dimensions with diameters range of 500 to 1000 nm and lengths between 5 and 20 μm. The other type of whisker was synthesized by using Y_2_O_3_ as the sintering aid and marked as Y_2_O_3_+Si_3_N_4_. The low-magnification SEM image included in Figure 1b shows the similarly smooth surfaces and lengths but smaller diameters (300–500 nm) of Y_2_O_3_+Si_3_N_4_ whiskers compared to the MgSiN_2_+Si_3_N_4_ whiskers.

### 2.3. In Situ SEM Nanomechanical Characterizations

For the Si_3_N_4_ bulk sample, the nanoindentation tests were conducted using a Hysitron Bruker™ PI 88 SEM PicoIndenter system (Billerica, MA, USA) inside a field emission high-resolution SEM (JEOL™ JSM-IT500HR/LA, Tokyo, Japan). The indentation experiments were conducted under load-controlled mode, with the maximum peak load set between 1400 and 6000 μN using a gradient distribution strategy. The loading and unloading durations were both set to 20 s. After reaching the peak load, the load was held for 10 s to allow the sample to relax and stabilize. A multi-point continuous testing mode was used, selecting a region on the flat area of the sample to set a 4 × 5 indentation array (a total of 20 points), with a 5 μm distance between points to ensure no mutual interference. After the tests, the modulus and hardness at each point were derived from the load-depth curves recorded by the equipment using the classic Oliver–Pharr model [36].

**Figure 2:**
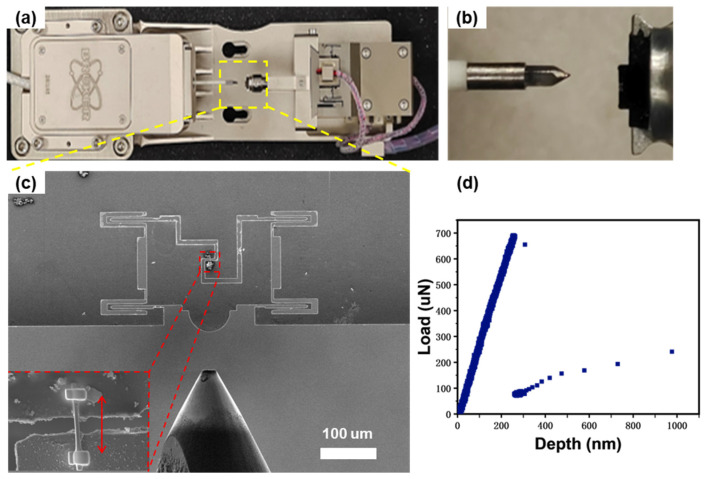
Overview of the in situ SEM whisker tensile testing platform: (**a**) photo of the PI 88 SEM PicoIndenter system; (**b**) magnified view of the yellow dotted box in figure (**a**); (**c**) SEM image of the PTP device; (**d**) typical load-displacement curve.

For Si_3_N_4_ whiskers, the Si_3_N_4_ whiskers were clamped onto a push-to-pull (PTP) micromechanical device and then subjected to tensile tests actuated by a picoindenter (Hysitron^TM^ PI 88 SEM PicoIndenter system) inside a high-resolution field-emission SEM (JEOL^TM^ JSM-IT500HR/LA). First, an individual whisker was placed vertically across the PTP device gap with the assistance of a sharp tungsten microneedle; subsequently, the as-transferred whisker was firmly fixed onto the device by depositing Pt at its two ends using the gas injection system (GIS) of a focus ion beam (FIB) machine. As shown in Figure 2c, the ‘pushing’ load actuated from the picoindenter and exerting onto the sphere device head could be transformed into the ‘pulling’ force across the device gaps, thus achieving the tensile test of the clamped Si_3_N_4_ whisker. As shown in the inset of Figure 2c, the Si_3_N_4_ whisker was clamped perpendicularly to the device gap with its ends fully embedded into the Pt solder paste, which ensures a uniaxial tensile test without whisker sliding. After loading the device onto the sample stage of the PI88 picoindenter and installing the indenter into the SEM, the in situ SEM tensile tests on individual Si_3_N_4_ whiskers can then be achieved by operating the TriboScan^TM^ v10 software controller equipped for PI 88.

The in situ SEM tensile tests of Si_3_N_4_ whiskers were conducted under displacement control mode with a displacement rate of 10 nm s^−1^ inside the SEM with an accelerating voltage of 10 kV. Figure 2a,b show the optical images of PI 88 SEM PicoIndenter stage and its indenter part. The PTP device was loaded counter to the indenter (Figure 2c). During the tensile test, the mechanical data of the tensile test could be read and recorded by the Perfermech^TM^ transducer of the PI88 picoindenter, while the deformation process could be directly monitored and filmed by the CCD camera equipped on the IT 500 FESEM. The force vs. displacement curve was listed in Figure 2d; from it we can see that, at the beginning, the load rose linearly with the increase of the indentation displacement, and this was followed by an abrupt load drop indicating the fracture of the whisker. Since the load before fracture contains both parts acting on the device and the sample, the suddenly dropped value reflects the portion acting on the sample, i.e., the strength of the whisker. Based on this, the fracture stress of the whisker specimen can be calculated. By analyzing the video frames recorded during the tensile tests, together with the determined sample gauge length, the fracture strain can then be measured.

## 3. Results and Discussion

### 3.1. In Situ Nanoindentation Testing

**Figure 3:**
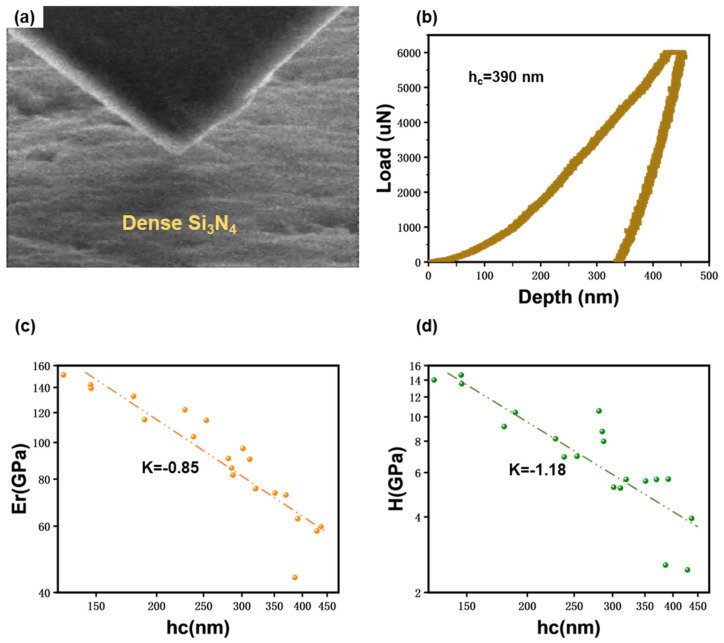
(**a**) Overview of the in situ SEM nanoindentation testing. (**b**) The load-depth curve. Variation with contact depth h_c_ of (**c**) reduced modulus and (**d**) indentation hardness of dense Si_3_N_4_ from indentation tests.

As shown in Figure 3a, the mechanical properties of bulk Si_3_N_4_ were studied by nanoindentation. Figure 3b shows the load vs. depth curve obtained from a representative nanoindentation test with a peak load set at 6000 μN. According to the Oliver–Pharr model [36,37], the contact depth can be expressed by Equation (1):

(1) $$h_{c}=h_{max}-\epsilon \frac{P_{max}}{S}$$

where $h_{max}$ is the maximum load depth; ϵ is a constant related to the indenter geometry, with a value of 0.72 for the conical indenter used in the test; $P_{max}$ is the maximum load; and S is the unloading contact stiffness. It should be noted that the contact stiffness is calculated only from the linear portion of the unloading curve, resulting in a contact depth of 390 nm.

The contact area under peak load is determined by the geometry of the indenter and the contact depth, as given by Equation (2):

(2) A=Fhc=C0hc2+C1hc+C2hc12+C3hc14+⋯+C8hc1128

where $h_{c}$ is the contact depth and $C_{0}$ to $C_{8}$ are fitting constants. The calculated contact area is A=1061634.34 nm^2^.

Hardness is given by Equation (3):

(3) $$H=\frac{P_{max}}{A}$$

the calculated hardness is H=5.65 GPa.

The modulus is given by Equation (4):

(4) $$E_{r}=\frac{\sqrt{\pi }}{2S\sqrt{A}}$$

the calculated modulus is $E_{r}=62.81$ GPa.

The figure clearly shows that during the initial loading, the material underwent recoverable elastic deformation; as the load increased, the indenter depth increased rapidly until the load reached a certain value, after which the depth increase rate slowed down. Upon reaching the peak load, the indenter was held for 10 s, during which the indenter depth continued to increase due to the creep. In the final unloading stage, the elastic deformation was recovered while the permanent plastic deformation remained.

The indentation hardness and elastic moduli as measured from 20 different sites at different indentation depths are listed in Figure 3c,d. It is worth noting that, although it is not included in the Oliver–Pharr model, the incorporation of the peak-load-holding step would enable a reliable unloading process, thus it was adopted in our experiments. The data show that both the elastic modulus and indentation hardness of the bulk Si_3_N_4_ exhibited a pronounced “the shallower, the stronger” indentation size effect. With the maximum indentation depth increasing from 125 to 450 nm, the elastic modulus and hardness reduced from 151.1 to 43.8 GPa and 14.7 to 2.5 GPa, respectively. By performing linear fitting, together with the power-law relationships E∝h_c_^K^ and H∝h_c_^K^, the contact-depth-dependent factors for reduced modulus and indentation hardness were fitted as −0.85 (Figure 3c) and −1.18 (Figure 3d), respectively, which indicated a strong indentation depth sensitivity of the Si_3_N_4_ bulk’s reduced modulus and indentation hardness. In particular, the indentation hardness was more sensitive. This may be because, compared to the direct influence of dislocations on indentation hardness, the modulus tends to approach the properties of bulk material as the contact depth increases, thus showing relatively less dependency on contact depth.

The greatest elastic modulus and indentation hardness of the bulk Si_3_N_4_ measured in our work were 151.1 GPa and 14.7 GPa, respectively, which were comparable to the previously reported results [38].

**Figure 4:**
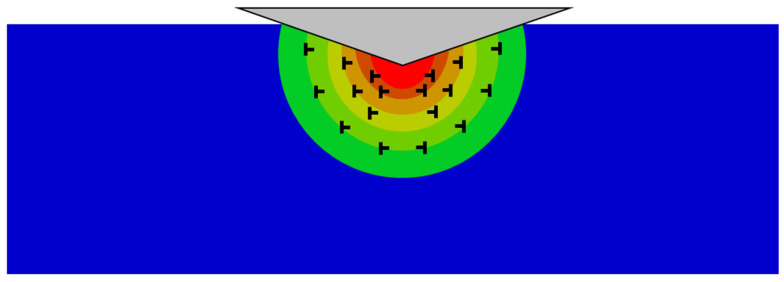
Diagram of the nanoindentation strain gradient.

Upon indentation, the penetration of the sharp cube corner tip will induce plastic deformation in a local area around the tip. To accommodate the strain inhomogeneity caused by local plastic deformation near the sample surface and avoid fracture, geometrically necessary dislocations (GND) will be generated at the edge of the deformation zone [39,40,41]. As the indenting goes on, these GNDs will further evolve into dislocation loops evenly distributing around the indentation dent, as shown in Figure 4. Therefore, taking columns surrounding the indenter tip as objects, the larger the column, the lower its dislocation density. This also means that the farther away from the indenter tip, the lower the dislocation density, which will inevitably induce a strain gradient around the dent. This phenomenon can explain the observed depth effect on the strength of the Si_3_N_4_ bulk. At small indentation depth, the nucleation of GNDs will induce considerably high strain hardening rates and finally lead to remarkably high strength. When the indentation depth is deepened, the nucleation of GNDs will be replaced by their glide and multiplication activities, which require less stress to promote further deformation. In addition, as the dislocation loops expand, the required stress driving them to glide will reduce, these together lead to a diminishing work hardening rate. Therefore, with the increase of the indentation depth, the measured strength or the apparent hardness of the Si_3_N_4_ will decrease. The same mechanism is also accountable for the measured depth-sensitive elastic modulus [42,43].

In addition to strain gradient plasticity theory, the geometrically necessary dislocations generated after nanoindentation interact with grain boundaries and statistically stored dislocations, leading to additional hardening effects [42]. These interactions are influenced by their associated characteristic dimensions, including the depth of the nanoindentation and grain size.

### 3.2. In Situ SEM Tensile Test

**Figure 5:**
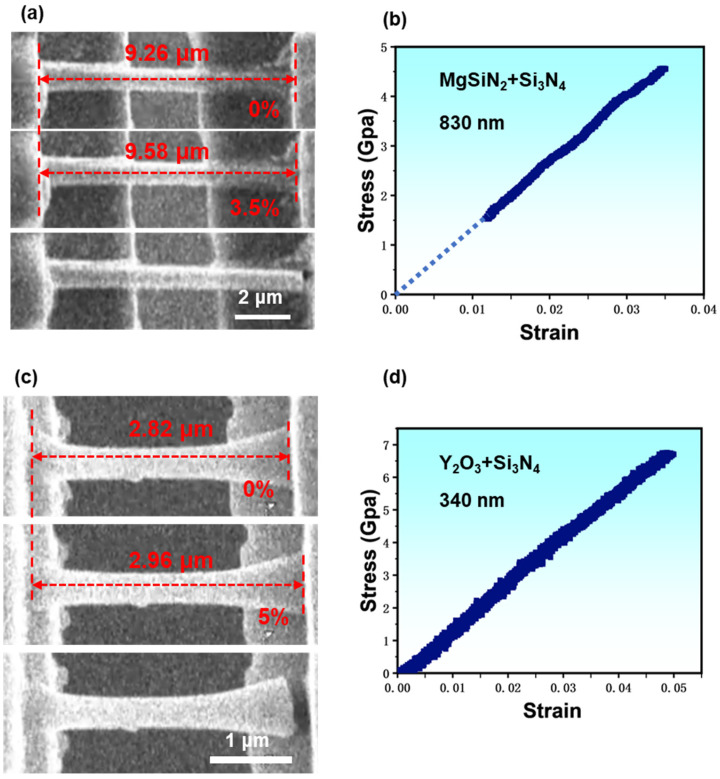
(**a**,**b**) Typical in situ SEM tensile test of a Mg+Si_3_N_4_ whisker with a diameter of 830 nm. (**a**) Sequential images recorded during in situ SEM tensile tests. The pristine whisker is shown at the top, and followed by the image before the fracture, while the bottom image shows the fractured whisker. (**b**) The stress-strain curve corresponding to (**a**). (**c**,**d**) Typical in situ SEM tensile test of a Y_2_O_3_+Si_3_N_4_ whisker with a diameter of 340 nm. (**c**) Sequential images recorded during in situ SEM tensile tests. The pristine whisker is shown at the top, and followed by the image before the fracture, while the bottom image shows the fractured whisker. (**d**) The stress-strain curve corresponding to (**c**).

Performing tensile tests is the optimal way to evaluate a solid material’s intrinsic mechanical properties. Due to limitations like precise microscale manipulation and high-resolution loading control, it is still challenging to perform uniaxial tensile tests on micro whiskers quantitatively as people did for macro dog-bone samples. Here, with the assistance of a high-resolution micromanipulator and a delicate picoindenter, we had managed to carry out quantitative tensile tests for individual Si_3_N_4_ whiskers inside SEM in an in situ manner. Such in situ tests inside the SEM on one hand allow the real-time monitoring of the sample’s deformation process; meanwhile, it could also enable the collecting of accurate mechanical data synchronously and correlate them together for further analysis. Typical tensile test results for the two different kinds of Si_3_N_4_ whiskers are shown in Figure 5. Figure 5a,b show the elongation and fracture process of a MgSiN_2_+Si_3_N_4_ whisker alongside its engineering stress vs. strain curve (see Video S1), and Figure 5c,d present the corresponding results of a Y_2_O_3_+Si_3_N_4_ whisker (see Video S2).

**Figure 6:**
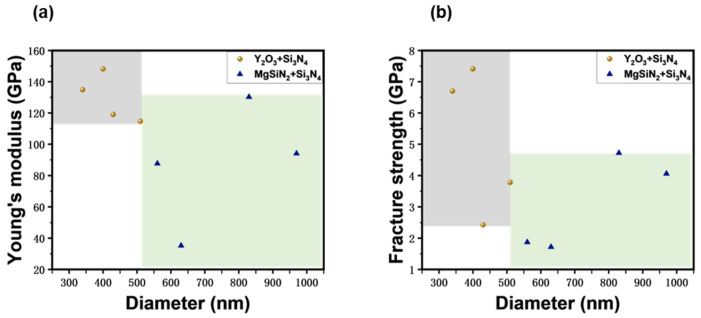
Variation with diameter of (**a**) Young’s modulus and (**b**) fracture strength of Si_3_N_4_ whiskers from in situ SEM tension tests.

In Figure 5a, showing the elongation process of a MgSiN_2_ sintering-aided Si_3_N_4_ whisker with a diameter of ~830 nm, we can see that the clamped whisker was gradually stretched to a strain of ~3.5% before fracture, and the fracture strength was calculated as ~4.56 GPa. Since no necking occurred during the entire stretching process and because of the almost linear responsiveness between the stress and strain (Figure 5b), we assume that the deformation process of the MgSiN_2_+Si_3_N_4_ whisker was dominated by elastic deformation, which was also evidenced by the typical brittle fracture morphology, as listed in the bottom image of Figure 5a. Therefore, we could directly calculate Young’s modulus simply by dividing the fracture stress by fracture strain, and it was ~130.29 GPa. In total, we stretched four MgSiN_2_+Si_3_N_4_ whiskers. The results are listed in Table 1. By comparing the mechanical data according to their whisker diameters, no obvious size dependence was found, as demonstrated by the solid blue triangles presented in Figure 6. Figure 5c lists the deformation process of a typical Y_2_O_3_+Si_3_N_4_ whisker with a diameter of ~340 nm, which is almost identical to the MgSiN_2_+Si_3_N_4_ whisker except a larger fracture strain. The corresponding stress vs. strain plot in Figure 5d also exhibits linearity with fracture stress and strain of ~6.7 GPa and 5%, respectively, and the corresponding Young’s modulus was calculated as ~134.89 GPa. The four Y_2_O_3_+Si_3_N_4_ whiskers stretched in our work are listed in Table 1 and presented in Figure 6 as well, represented by brown dots. From Figure 6 we can also see that neither the Young’s modulus nor the fracture strength of the Y_2_O_3_+Si_3_N_4_ whiskers show visible size dependence.

We selected four Si_3_N_4_ whiskers from each of the two types for in situ uniaxial tensile testing to analyze the effect of sintering aids on the mechanical properties of the whiskers. The MgSiN_2_+Si_3_N_4_ whiskers have diameters between 500 and 1000 nm, while the Y_2_O_3_+Si_3_N_4_ whiskers have diameters between 300 and 500 nm. The test results are shown in Figure 6. Figure 6a shows the calculated Young’s modulus as a function of diameter for all tested whiskers, while Figure 6b shows the fracture strength as a function of size. In Figure 6, the gray box represents the range of test values for Y_2_O_3_+Si_3_N_4_ whiskers, while the green box represents the range for MgSiN_2_+Si_3_N_4_ whiskers. 

In Table 1, we summarize the mechanical data measured on the two types of Si_3_N_4_ whiskers. Through comparison, it is easy to find that the modulus and strength of Y_2_O_3_+Si_3_N_4_ whiskers are higher than those of MgSiN_2_+Si_3_N_4_ whiskers, and the Young’s modulus and fracture strength of both types of whiskers show no significant dependence on size (See also Figure 6). The maximum tested Young’s modulus for MgSiN_2_+Si_3_N_4_ whiskers is 130.29 GPa with a diameter of 830 nm, and the maximum fracture strength at the same diameter is 4.56 GPa. For Y_2_O_3_+Si_3_N_4_ whiskers, the maximum tested Young’s modulus is 148.18 GPa with a diameter of 400 nm, and the maximum fracture strength at the same diameter is 7.41 GPa. Unlike metallic materials, which tend to exhibit a “smaller is stronger” trend at the micro–nano scale due to mechanisms like changes in dislocation sources [44], the fracture strength of brittle ceramic materials usually shows significant dispersion. This phenomenon can be analyzed using Griffith’s theory. According to Griffith’s micro-crack theory [45], a fracture originates from the most critical crack in the material, and the fracture strength is related to the size of the most critical crack in the region under tensile stress. Due to the random distribution of inherent cracks in the material, the size of the most critical crack varies among different samples, and its location is also randomly distributed. Hence, the fracture strength of the material exhibits statistical dispersion. In 1951, Swedish scholar Weibull proposed the Weibull distribution function to describe the fracture distribution in brittle materials [46], which could be applied here to describe the randomness of our measured mechanical data for Si_3_N_4_ whiskers.

The difference in mechanical properties between the two types of whiskers is presumably caused by their different fabrication method, i.e., different sintering aids. The Si_3_N_4_ whiskers synthesized with Y_2_O_3_ sintering aid generally have higher modulus and strength than those synthesized with MgSiN_2_ sintering aid. The sintering aids may affect the modulus and strength of the Si_3_N_4_ whiskers by controlling the microstructure. Additionally, the Si_3_N_4_ whiskers synthesized with Y_2_O_3_ sintering aid have smaller diameters. As the size decreases, the number of defects correspondingly reduces, increasing the fracture strength. Figure 6a shows that the Si_3_N_4_ whiskers synthesized with MgSiN_2_ sintering aid exhibit no size dependence on Young’s modulus and are relatively scattered. This may be because their diameters are too large, making their properties close to bulk materials, thus lacking size dependence on the modulus. In contrast, the Si_3_N_4_ whiskers synthesized with Y_2_O_3_ sintering aid show less dispersion in Young’s modulus, and the modulus values are generally higher than those of MgSiN_2_+Si_3_N_4_ whiskers. Besides the influence of the sintering aid, the smaller diameter of the Si_3_N_4_ whiskers synthesized with Y_2_O_3_ sintering aid result in a higher surface-to-volume ratio. This increased surface elasticity leads to a higher modulus [47], a phenomenon observed in other covalent crystal nanowires, such as Si nanowires [48,49] and SiC nanowires [50].

## 4. Conclusions

In summary, we conducted in situ mechanical investigations on Si_3_N_4_, bulk and whiskers, using SEM. For bulk Si_3_N_4_, 20 points of indentation tests with gradually increased indentation depths from 125 to 450 nm exhibited a strong depth-dependent trend. The elastic modulus and hardness increased as the contact depth decreased, with maximum values reaching 151.05 GPa for Young’s modulus and 14.67 GPa for the hardness. Statistical analysis of all data revealed a consistency between the elastic modulus and hardness, indicating that as hardness increases at smaller contact depths, the corresponding elastic modulus also increases. For Si_3_N_4_ whiskers synthesized with different sintering aids, in situ tensile tests showed that Si_3_N_4_ whiskers synthesized with Y_2_O_3_ sintering aid had higher Young’s modulus and fracture strength than those synthesized with MgSiN_2_ sintering aid. This difference is attributed not only to the impact of the sintering aid on the microstructure but also to the different whisker sizes produced by different sintering aids, with smaller sizes exhibiting stronger performance. These findings provide valuable insights into the intrinsic mechanical properties of Si_3_N_4_, which are critical for the future design and optimization of advanced silicon-nitride-based composites. Moreover, this work lays the foundation for further investigations into different mechanical testing methods such as buckling, three-point bending, and fatigue testing. Additionally, future studies could explore the effects of other sintering aids and expand the research to other material systems, thereby further advancing the understanding and application of Si_3_N_4_ materials in both scientific and industrial contexts.

## Figures and Tables

**Table 1:** The diameter and corresponding fracture properties from the eight samples.

	Diameter (nm)	Young’s Modulus (GPa)	Fracture Strength (GPa)	Fracture Strain	Sintering Aid
1	340	134.89	6.7	0.05	Y_2_O_3_
2	400	148.18	7.41	0.05	Y_2_O_3_
3	430	119.073	2.422	0.02	Y_2_O_3_
4	510	114.72	3.78	0.033	Y_2_O_3_
5	560	87.66	1.87	0.021	MgSiN_2_
6	630	35.23	1.72	0.049	MgSiN_2_
7	830	130.29	4.56	0.035	MgSiN_2_
8	970	94.05	4.06	0.043	MgSiN_2_

## Data Availability

The original contributions presented in the study are included in the article/Supplementary Material, further inquiries can be directed to the corresponding authors.

## Supplementary Materials

The following supporting information can be downloaded at: https://www.mdpi.com/article/10.3390/ma17184549/s1. Video S1 and Video S2.
